# A novel Bruton's tyrosine kinase inhibitor JDB175 shows potent efficacy to suppress central nervous system lymphoma

**DOI:** 10.1002/mco2.424

**Published:** 2023-11-04

**Authors:** Yong Xia, Xue Li, Ning Jiang, Xiawei Wei

**Affiliations:** ^1^ Laboratory of Aging Research and Cancer Drug Target State Key Laboratory of Biotherapy and Cancer Center National Clinical Research Center for Geriatrics West China Hospital Sichuan University Chengdu China; ^2^ Jumbo Drug Bank Co., Ltd., High‐Tech Zone Chengdu China

**Keywords:** Bruton's tyrosine kinase, central nervous system, JDB175, lymphoma

## Abstract

Patients with central nervous system (CNS) lymphoma face limited treatment options and poor treatment outcomes, emphasizing the urgent need for effective therapeutic strategies. One limiting factor contributing to the suboptimal efficacy is the inadequate penetration of most treatment drugs across the blood–brain barrier (BBB). Recent insights into the pathophysiology of CNS lymphoma have identified the Bruton's tyrosine kinase (BTK) signaling pathway as a potential target. Some clinical trials have shown impressive responses to BTK inhibitors in CNS lymphoma. However, currently approved BTK inhibitors have low BBB penetration rates, limiting their efficacy. In this study, we discovered that JDB175, a novel and highly selective BTK inhibitor, exhibits excellent BBB penetration capabilities and demonstrates favorable activity in a mouse model of CNS lymphoma while showing no significant signs of toxicity. JDB175 effectively inhibits the BTK signaling pathway in human lymphoma cells, suppressing their proliferation, inducing cell cycle arrest, and promoting apoptosis. The significance of this study lies in addressing the critical unmet medical need for effective treatments for CNS lymphoma. This finding indicates a promising avenue for improved treatments in CNS lymphoma, potentially opening doors for further clinical investigation and therapeutic advancements.

## Graphical abstract

Currently approved Bruton's tyrosine kinase (BTK) inhibitors have limited blood–brain barrier (BBB) permeability, which restricts their efficacy in treating central nervous system (CNS) lymphoma. JDB175, a novel, highly selective, and active BTK inhibitor, demonstrates excellent BBB penetration in rats. It effectively inhibits the BTK signaling pathway in human lymphoma cells, suppressing their proliferation, inducing cell cycle arrest, and promoting apoptosis in vitro. Furthermore, it exhibits promising activity in a mouse model of CNS lymphoma while showing no significant signs of toxicity. Further clinical trials are necessary to investigate its therapeutic efficacy in treating CNS lymphoma.

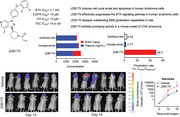

## 1 INTRODUCTION

Central nervous system (CNS) lymphoma is a form of non‐Hodgkin lymphoma in which cancer cells from lymph tissue form in the CNS (primary CNS lymphoma, PCNSL) or spread from other parts of the body to the CNS (secondary CNS lymphoma, SCNSL).^1^, ^2^ Most PCNSLs are typically diffuse large B‐cell lymphomas (DLBCLs). SCNSL is typically characterized by DLBCL and less common types of SCNSL include Burkitt lymphoma and T‐cell lymphoma.^3^ CNS involvement is typically a catastrophic event and patients with PCNSL and SCNSL have dismal outcomes.^1^, ^4^ The availability of clinical trials in patients with CNS lymphoma is limited, and enrolling patients with CNS lymphoma can be challenging, which puts CNS lymphoma patients in a worse situation.^5^ Patients with relapsed or refractory PCNSL exhibit limited responses to the majority of available therapies due to the presence of the blood–brain barrier (BBB).^6^ The standard of care for CNS lymphoma includes chemotherapy, autologous stem cell transplant, and whole‐brain radiotherapy.^7^, ^8^ However, these therapies require hospital admission and are highly toxic, which limits effective treatment for many patients, especially the elderly with poor health conditions or comorbidities.^9^, ^10^ Therefore, more effective drugs that have better infiltration ability across the BBB with lower toxicity are urgently needed.

PCNSL relies on chronically active B‐cell receptor (BCR) signaling.^11^ Bruton's tyrosine kinase (BTK) is a non‐receptor kinase that has been identified as an essential component of the BCR signaling pathway. It plays pivotal roles in lymphoma, particularly B‐cell lymphomas.^12^ It facilitates the transmission of signals from the BCR, promoting B‐cell proliferation and survival. Dysregulation of BTK can lead to uncontrolled growth of B cells, contributing to lymphomagenesis. Additionally, the role of BCR in recognizing antigens and initiating signaling pathways makes it a central player in the development of B‐cell lymphomas, as chronic BCR stimulation can lead to aberrant B‐cell proliferation. Consequently, targeting BTK has become a promising therapeutic approach in the treatment of various B‐cell lymphomas, offering the potential to disrupt these pro‐survival signals and inhibit lymphoma progression. As a result, there is currently a considerable interest in developing BTK inhibitors to treat lymphoma.^12^, ^13^

Currently, several small molecule inhibitors of BTK, such as ibrutinib, acalabrutinib, and zanubrutinib, have been approved for the treatment of certain types of lymphoma that have relapsed and/or have not responded to other therapies.^14^ Ibrutinib was the pioneering BTK inhibitor to receive approval, significantly altering the standard‐of‐care treatment for various cancers, including mantle cell lymphoma, marginal zone lymphoma, and several others.^6^ It is a first‐generation BTK inhibitor that has shown excellent anti‐cancer activities in different subtypes of B‐cell lymphomas.^15^ Recently, it has shown superior survival compared with BBB‐penetrating chemotherapy in lymphoma patients with CNS involvement, indicating the promising potencies of BTK inhibitors to treat CNS lymphoma.^16^ However, brain exposure to ibrutinib and two other approved BTK inhibitors is low, and the penetration rates (%, *C*_max,brain_/*C*_max,plasma_) are all below 10%, which may hinder their efficacy in treating CNS lymphoma.^17^ Therefore, the development of new small molecule inhibitors with strong BTK inhibition and robust BBB penetration capability holds significant clinical value.

In this study, we present a novel and highly selective BTK inhibitor called JDB175, which is a pyrazolopyridine compound with potent activities. We investigated its anti‐tumor activities and mechanisms in lymphoma. Given its excellent ability to penetrate the BBB, we established a preclinical model of CNS lymphoma to assess the efficacy of JDB175 in treating this condition. The data obtained demonstrated the strong inhibitory effects of JDB175 on the growth of lymphoma cells. Moreover, it exhibited notable activities in suppressing CNS lymphoma in mice. This study holds significant importance as it addresses the pressing need for more effective therapeutic strategies in the treatment of CNS lymphoma, a condition marked by limited treatment options and poor outcomes. The findings of this study underscore the potential of JDB175 as a therapeutic option, warranting further clinical investigations and offering hope to patients facing this challenging disease.

## 2 RESULTS

### 2.1 JDB175 suppressed the viabilities of lymphoma cells in vitro

**FIGURE 1:**
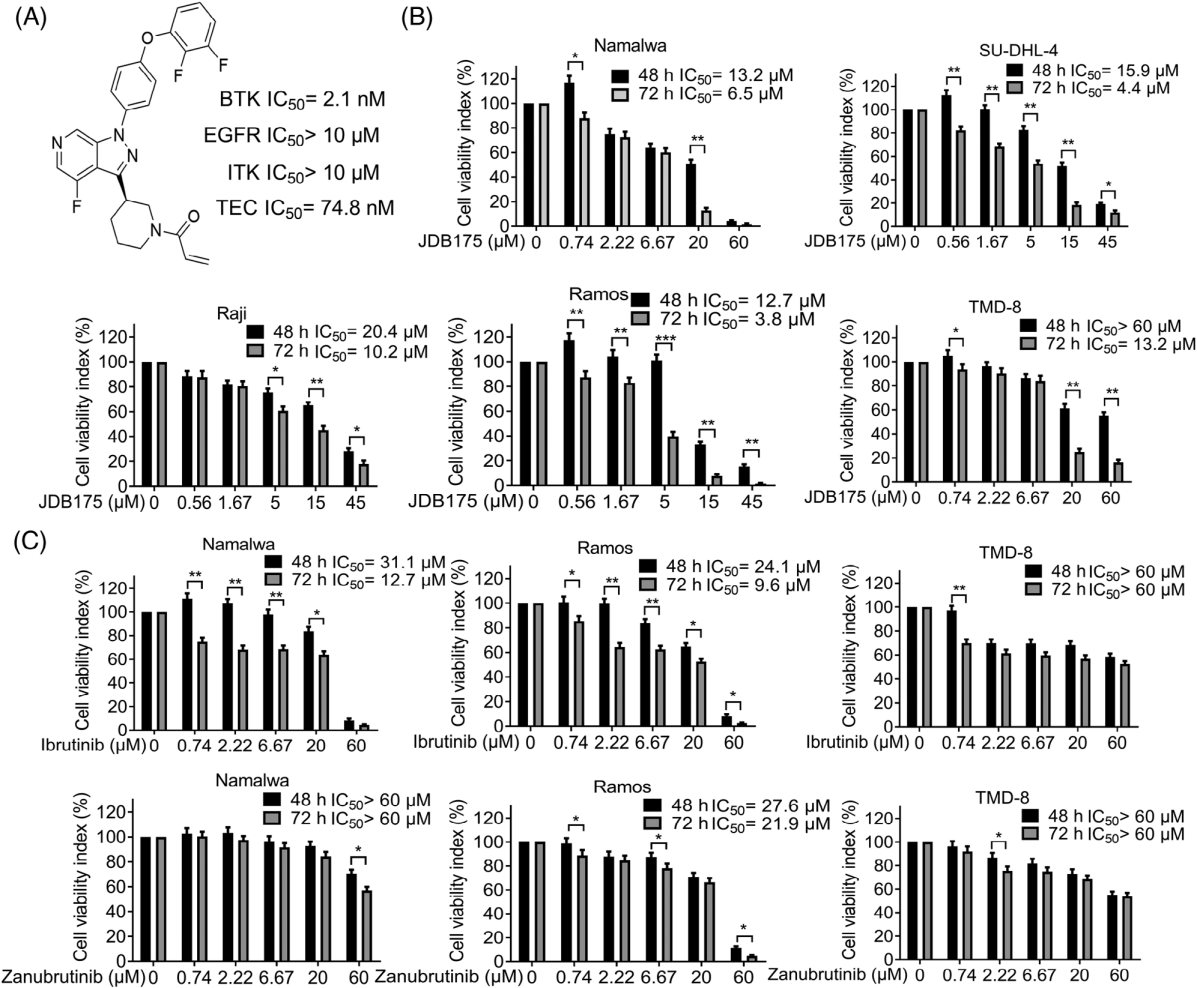
JDB175 suppresses lymphoma cell viability. (A) The chemical structure of JDB175 and its inhibitory activities against BTK, EGFR, ITK, and TEC. (B) Lymphoma cells were treated with varying concentrations of JDB175 for 48 and 72 h, followed by assessing cell viability using the cell counting kit‐8 (CCK‐8 assay. Inhibition rates were calculated, and IC_50_ values (µM) were determined. (C) Namalwa, Ramos, and TMD‐8 cells were treated with varying concentrations of ibrutinib and zanubrutinib for 48 and 72 h, respectively. Cell viability was subsequently determined using the CCK‐8 assay. Inhibition rates were calculated, and IC_50_ values (µM) were derived. ^*^ *p* < 0.05, ^**^ *p* < 0.01 compared to the vehicle treatment group.

The chemical structure of JDB175 is depicted in Figure 1A. The kinase inhibitory activities of JDB175 against a panel of recombinant human protein kinases were assessed using a radiometric kinase assay. Notably, JDB175 exhibited potent inhibition of BTK, with an IC_50_ value of 2.1 nM. Importantly, it showed no or minimal inhibition of epidermal growth factor receptor (EGFR), IL‐2‐inducible T‐cell kinase (ITK), and TEC (Figure 1A), which are known to cause adverse effects associated with BTK inhibitors such as ibrutinib.^18^ Furthermore, JDB175 demonstrated almost no inhibitory activity against the other 94 selected protein kinases. To investigate the effects of JDB175 on lymphoma cell viability, five lymphoma cell lines were treated with JDB175 and the inhibition rates were calculated. The data demonstrated that JDB175 exerted a time‐ and dose‐dependent inhibition on their cell viabilities (Figure 1B). After 48 h of treatment, the IC_50_ values were below 20 µM in four cell lines, and they decreased further after an additional 24 h of treatment. Ibrutinib and zanubrutinib are two approved BTK inhibitors, and we also assessed the activity of these two BTK inhibitors in Namalwa, Ramos, and TMD‐8 cells. As shown in Figure 1C, JDB175 demonstrated superior activity in inhibiting lymphoma cell viability compared to ibrutinib and zanubrutinib. In summary, these findings indicated that JDB175 is a novel and highly selective BTK inhibitor that exhibited potent suppression of lymphoma cells.

### 2.2 JDB175 induced G0/G1 phase arrest in lymphoma cells

**FIGURE 2:**
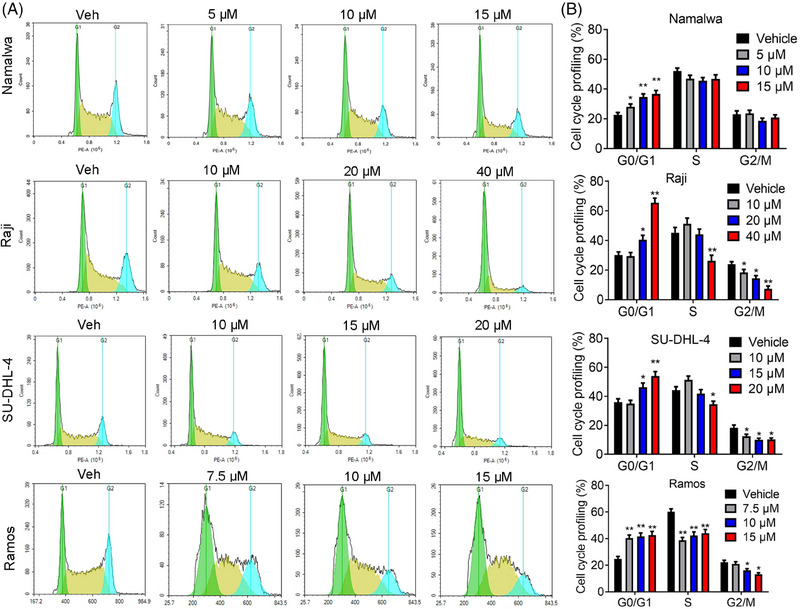
JDB175 induces G0/G1 arrest in lymphoma cells. (A) Four types of lymphoma cells were treated with varying concentrations of JDB175 for 48 h, collected, and fixed in 75% ethanol for 24 h. Subsequently, they were incubated with a buffer containing 50 µg/mL propidium iodide (PI), 50 µg/mL RNase A, and 0.1% Triton X‐100 for 30 min. Cell cycle distribution was then determined using fluorescence flow cytometry (FCM). (B) Quantification of the cell cycle distribution in (A). ^*^ *p* < 0.05, ^**^ *p* < 0.01 versus vehicle.

The inhibitory effects of JDB175 on lymphoma cells may be attributed to the induction of cell death and/or cell cycle arrest. Dysregulated cell cycle progression is a hallmark of cancer and a potential target for anti‐tumor therapy. To explore the underlying mechanisms of JDB175's inhibitory effects, we evaluated its impact on cell cycle distribution. As depicted in Figure 2A,B, treatment with 15 µM JDB175 for 48 h significantly increased the G0/G1 phase distribution from 22.6% in the vehicle‐treated group to 36.7% in Namalwa cells. Similar observations were made in Raji, SU‐DHL‐4, and Ramos cells. These data demonstrated that JDB175 prominently arrested lymphoma cells in the G0/G1 phase, contributing to its anti‐lymphoma effects.

### 2.3 JDB175 induced apoptosis of lymphoma cells

**FIGURE 3:**
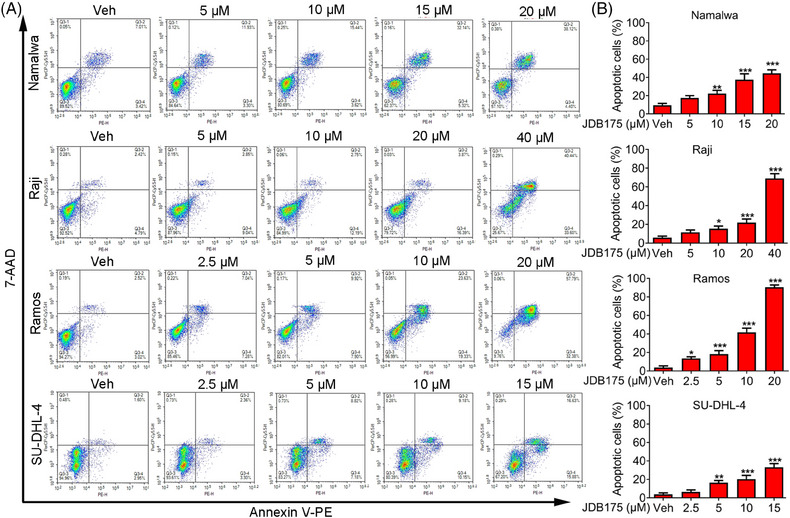
JDB175 induces apoptosis in lymphoma cells. (A) After a 72‐h treatment with specified concentrations of JDB175, the cells were harvested and delicately washed with phosphate‐buffered saline (PBS). Subsequently, they were individually stained with Annexin V‐PE and 7‐AAD, followed by a 15 min incubation at room temperature in dark conditions. Cell apoptosis rates were then determined using an ACEA NovoCyte flow cytometer and subsequently analyzed utilizing NovoExpress software. (B) Quantification of apoptosis rates in (A). ^*^ *p* < 0.05, ^**^ *p* < 0.01, ^***^ *p* < 0.001 versus vehicle.

The decrease in cell viabilities following JDB175 treatment may also be attributed to the induction of apoptosis. Thus, we examined the effects of JDB175 on apoptosis in lymphoma cells. As shown in Figure 3A,B, JDB175 induced concentration‐dependent apoptosis in all four lymphoma cell lines after 72 h of treatment. At concentrations of 20 and 40 µM, it induced apoptosis in more than 50% of Ramos and Raji cells, respectively, indicating robust apoptosis‐inducing effects. In summary, these data suggested that the induction of apoptosis in lymphoma cells is another mechanism by which JDB175 exerts its anti‐cancer effects.

### 2.4 The effects of JDB175 on multiple key signaling pathways in lymphoma cells

**FIGURE 4:**
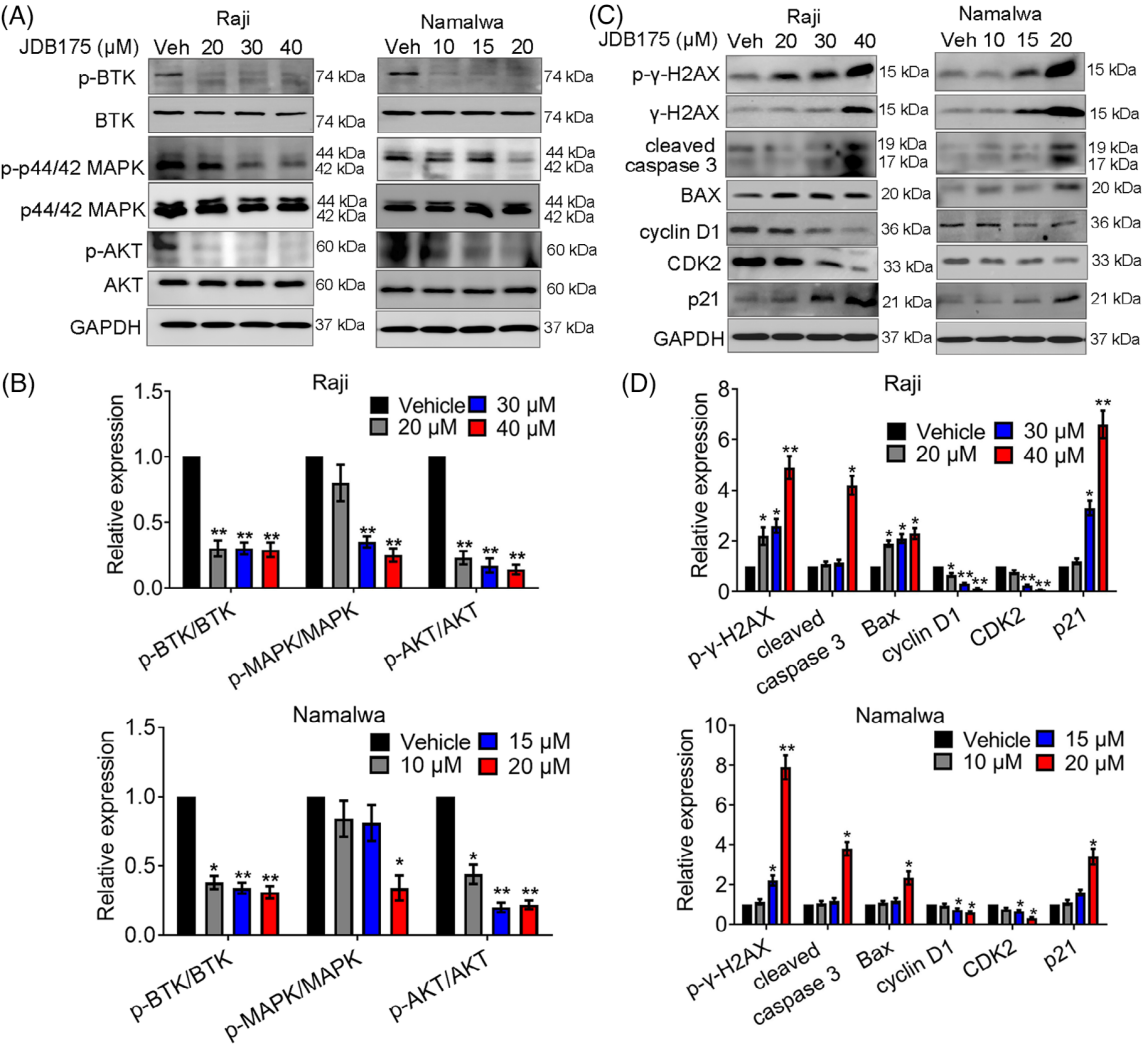
Effects of JDB175 on multiple signaling pathways in lymphoma cells. (A) Raji and Namalwa cells were treated with indicated concentrations of JDB175 for 48 h, followed by western blot analysis of p‐BTK, total BTK, p‐p44/42 MAPK, total p44/42 MAPK, p‐AKT, and AKT. (B) Quantification of signals of the bands from panel (A). MAPK refers to p44/42 MAPK in the figures. (C) The expressions of key proteins related to apoptosis and cell cycle regulation were examined by western blot analysis in the above cell lines after treatment with JDB175 for 48 h. (D) Quantification of blot signals from panel (C). ^*^ *p* < 0.05, ^**^ *p* < 0.01 versus vehicle.

Furthermore, western blot analysis was performed to assess the effects of JDB175 on key signaling pathways in Namalwa and Raji cells. As depicted in Figure 4A,B, JDB175 effectively suppressed BTK phosphorylation in both cell lines, with even the lowest concentrations nearly abolishing the phosphorylation. BTK is crucial for several active pathways involved in cancer cell survival, including protein kinase B (AKT) and p44/42 MAPK. Our data demonstrated that JDB175 also effectively inhibited the activation of AKT and p44/42 MAPK in Namalwa and Raji cells. These findings collectively indicated that JDB175 could effectively inhibit BTK and the downstream pathways in these cell lines.

Additionally, we assessed the effects of JDB175 on the expression of key proteins involved in apoptosis and cell cycle regulation in both cell lines. As shown in Figure 4C,D, JDB175 induced noticeable cleavage of caspase‐3, a pivotal event during apoptosis. It also upregulated the expression of Bax, a key pro‐apoptotic protein in the Bcl‐2 family. Furthermore, it increased the expression of both phosphorylated and total γ‐H2AX, indicating the induction of DNA double‐strand breaks. Moreover, we evaluated the expression of crucial regulators in cell cycle progression. JDB175 treatment downregulated the expressions of cyclin D1 and CDK2. P21, a key suppressor of the G1/S cell cycle transition, was upregulated by JDB175 in both cell lines. These findings were consistent with the effects observed in the flow cytometry (FCM) analysis and collectively demonstrated that JDB175 induced apoptosis and G0/G1 cell cycle arrest in lymphoma cells.

### 2.5 JDB175 showed potent inhibitory effects and good safety profiles in a CNS lymphoma model in vivo

**FIGURE 5:**
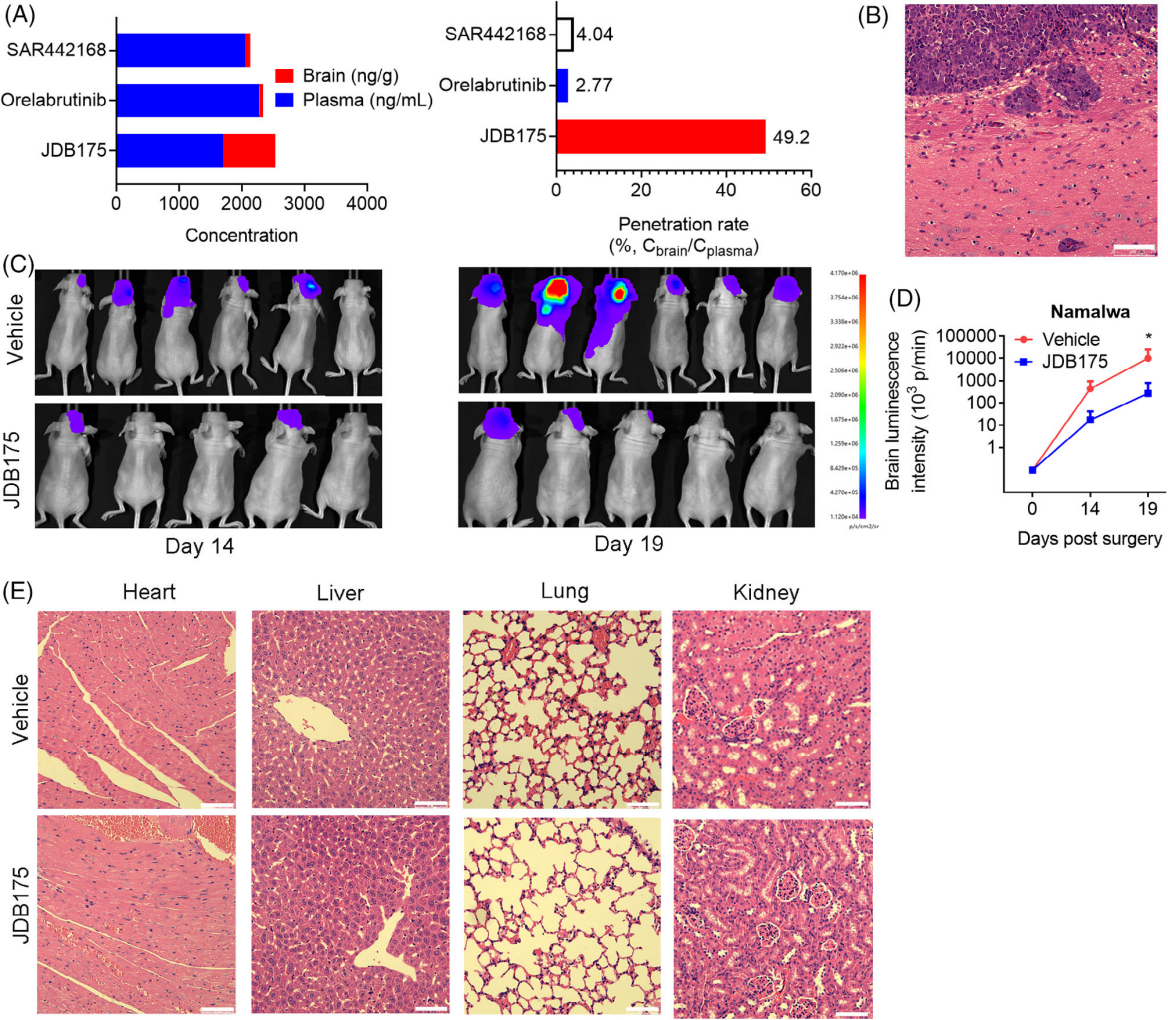
Anti‐cancer activity and preliminary safety of JDB175 in a murine central nervous system (CNS) lymphoma model. (A) JDB175, orelabrutinib, and tolebrutinib (SAR442168) were orally administered to Sprague–Dawley (SD) rats at a dose of 10 mg/kg, and their concentrations in both plasma and brain were determined by liquid chromatography–mass spectrometry (LC–MS) analysis at 0.5 h after administration. Permeability across the blood–brain barrier (BBB) at this time point was also calculated. (B–D) A murine CNS lymphoma model was established in nude mice by intracarotid injection of 5 × 10⁵ Namalwa cells expressing luciferase. The oral administration of JDB175 (150 mg/kg) commenced 3 days post‐surgery, with a dosing frequency of once daily. Thirty days after the oral administration of JDB175, mice in the vehicle‐treated group were sacrificed, and their brains were extracted for hematoxylin and eosin (H&E) staining to ascertain the presence of brain tumor. (B) Representative images of intracranial tumors stained with H&E. Scale bar is 75 µm. (C) In vivo imaging was performed to assess the luminescence intensity of intracranial tumors in different groups of mice at days 14 and 19 post‐surgery. (D) Quantitative results of intracranial tumor signal in the murine CNS lymphoma model. (E) Histological examination of the heart, liver, lung, and kidney of mice was conducted by H&E staining after treatment with JDB175 to preliminarily evaluate its potential toxicity to vital organs in mice. Scale bar is 75 µm. ^*^ *p* < 0.05 versus vehicle.

We are curious about the potent suppressive abilities of JDB175 on CNS lymphoma. BBB is a critical barrier for delivery of small molecules into the brain. Therefore, we assessed the abilities of JDB175 to penetrate the BBB and go into the brain of rat in vivo. As shown in Figure 5A, JDB175 was highly absorbed in the brain of rat after a single dose of 10 mg/kg. Following oral administration, at 0.5 h, JDB175 exhibited significantly high concentrations in both the blood and brain, reaching 1697 ng/mL and 835 ng/g, respectively. The brain penetration rate of JDB175 (%, *C*_brain_/*C*_plasma_) was also remarkably high at 0.5 h after oral administration, reaching 49.2%, surpassing the other two BTK inhibitors, orelabrutinib and tolebrutinib (SAR442168). These data demonstrated the robust potential of JDB175 to penetrate the BBB and exhibit promising abilities in inhibiting CNS lymphoma.

Subsequently, we established a CNS lymphoma model utilizing Namalwa cells, and confirmation of successful establishment was achieved through hematoxylin and eosin (H&E) staining of the brain (Figure 5B). As shown in Figure 5C,D, in vivo imaging data indicated that the tumor signal intensity of JDB175‐treated mice was weaker than that in the vehicle treatment group.

The aforementioned data indicated that JDB175 possesses the potential to serve as a novel therapeutic agent for treating CNS lymphoma. A favorable safety profile represents another crucial characteristic of a successful anti‐cancer drug. In this regard, we conducted preliminary assessments of JDB175's safety profile during in vivo experiments. Histological examinations of the heart, liver, kidney, and lung following H&E staining revealed no evident signs of pathological changes induced by JDB175 in these major organs (Figure 5E). In summary, these data suggested that JDB175 demonstrated potent inhibitory effects and exhibited a favorable safety profile in the in vivo Namalwa CNS lymphoma model.

## 3 DISCUSSION

CNS involvement poses a significant threat to the elderly or frail patients with lymphoma, with poor survival rates of less than 2 years, and even worse outcomes for those with recurrent/refractory disease.^13^ Despite recent therapeutic advances in the field, the current treatment options remain limited. These include polychemotherapy regimens based on high‐dose methotrexate, whole‐brain radiotherapy, and autologous stem cell transplant.^4^ However, achieving durable remission remains challenging. Moreover, these therapies have drawbacks, such as failure of salvage treatment, significant late neurotoxicity, and unsuccessful stem cell harvest.^19^ Additionally, there is a lack of clinical trial information available due to the exclusion of patients with CNS lymphoma from many clinical trials.^20^ These current limitations underscore the urgent need for novel therapeutic approaches.

Recent advancements in understanding the pathophysiology of CNS lymphoma have led to the identification of numerous potential therapeutic targets.^21^, ^22^ One such target is the BTK signaling pathway. PCNSL has been found to exhibit aberrant activation of BCR/nuclear factor‐κB signaling pathways, which are central signaling axes in PCNSL and potential targets for molecular therapy.^23^, ^24^ BTK inhibitors have been utilized in clinical trials for the treatment of CNS lymphoma and have shown promising and impressive responses.^25^, ^26^ The National Comprehensive Cancer Network guidelines also included BTK inhibitors as the therapeutic option for the treatment of relapsed/refractory (R/R) PCNSL in 2018.^27^ However, these BTK inhibitors have certain limitations, particularly in their ability to penetrate the BBB. A previous study has demonstrated low penetration rates (expressed as a percentage of *C*_max,brain_/*C*_max,plasma_) for approved BTK inhibitors such as zanubrutinib (3.5%), tirabrutinib (8.5%), and ibrutinib (9.8%).^17^ Therefore, there is an urgent need to develop highly efficient BTK inhibitors capable of crossing the BBB.

In this study, JDB175, a novel and highly selective orally administered BTK inhibitor, demonstrated strong BBB penetration capabilities. The brain penetration rate of JDB175 reached 49.2%, much higher than that of orelabrutinib and SAR442168 and far higher than that of zanubrutinib, tirabrutinib, and ibrutinib. JDB175 exhibited potent activity in inhibiting growth, inducing cell cycle arrest, and promoting apoptosis in human lymphoma cells in vitro. Additionally, it showed good inhibitory activity against the BTK signaling pathway and pathways associated with cell proliferation and cell cycle progression. Importantly, it exhibited potent inhibitory effects without obvious toxicities in a mouse model of CNS lymphoma. Inhibition of EGFR, ITK, and TEC could lead to untoward effects of BTK inhibitors, such as ibrutinib.^18^ Importantly, JDB175 showed little to no inhibition of their kinase activities, indicating its potential for high safety.

Resistance to BTK inhibitors limits the long‐term use and reduces progression‐free survival in B‐cell malignancies. Recent molecular insights into the genomic alterations driving PCNSL have revealed the involvement of additional target pathways that contribute to PCNSL development and resistance to treatment, including resistance to BTK inhibitors.^21^, ^22^ These pathways include the phosphatidylinositol‐3‐kinase (PI3K)/mammalian target of rapamycin (mTOR) pathway and immune evasion mechanisms. Various clinical trials have investigated the effects of small molecules targeting these pathways and immune checkpoint inhibitors, such as immunomodulatory imide drugs,^28^, ^29^ PI3K/AKT/mTOR inhibitors,^22^ and immune checkpoint inhibitors,^21^ for the treatment of CNS lymphoma. Some of these agents have shown promising therapeutic efficacy. For example, pembrolizumab, an anti‐PD‐1 antibody, has demonstrated encouraging outcomes, achieving complete response in three out of five patients, with progression‐free survival exceeding 13 months.^30^ Furthermore, chimeric antigen receptor T (CAR‐T) cell therapy has shown potential for treating CNS lymphoma, with anti‐CD19 CAR‐T cells yielding significant treatment responses in R/R PCNSL.^31^, ^32^ Combination therapy has been explored extensively in the treatment of CNS lymphoma, as it has the potential to improve efficacy and overcome or delay resistance. Numerous clinical trials have investigated the therapeutic potential of combining BTK inhibitors with other drugs for CNS lymphoma treatment, including chemotherapy agents, immune checkpoint inhibitors, PI3K/AKT/mTOR inhibitors, immunomodulatory imide drugs, and various multi‐agent combinations.^16^, ^33^ Given the superior BBB penetration of JDB175 compared to currently approved BTK inhibitors, it is essential to evaluate the activity of BTK inhibitors in combination with these agents in future clinical trials for the inhibition of CNS lymphoma.

Nonetheless, this study presents certain limitations. It relied solely on established lymphoma cell lines to assess both in vitro and in vivo inhibitory activities of JDB175. Future investigations should encompass the use of primary lymphoma cells derived from patients, especially when establishing CNS lymphoma models, in order to yield results of greater clinical relevance. Safety considerations represent an essential prerequisite for drug approval. In this study, preliminary safety assessments of JDB175 in mice were limited to H&E staining of vital organs, underscoring the need for more extensive and specialized testing to comprehensively evaluate its safety profile. Small molecule inhibitors often confront the challenge of post‐treatment resistance development. Proactive identification of potential resistance mechanisms and the exploration of strategies for reversing resistance can offer substantial benefits to patients, although this particular aspect remained unexplored in our study. Should JDB175 exhibit promising inhibitory effects on CNS lymphoma in early clinical trials, it becomes imperative to promptly initiate research into its resistance mechanisms.

In summary, this study has discovered that JDB175, a novel and highly selective BTK inhibitor, exhibits therapeutic activity against CNS lymphoma with excellent BBB penetration and favorable safety profile. It exerts strong inhibition of the BTK signaling pathway. Importantly, it exhibits a significantly higher penetration rate across the BBB compared to currently approved BTK inhibitors. In a mouse model of CNS lymphoma, JDB175 demonstrated good inhibitory effects while showing no significant signs of toxicity. In in vitro experiments, it effectively suppresses the proliferation of human lymphoma cells, induces cell cycle arrest, and promotes apoptosis. Therefore, it is imperative to explore the inhibitory potential of JDB175 as a monotherapy and in combination with other therapeutic approaches through clinical trials for CNS lymphoma. Such investigations hold the promise of offering novel treatment options to patients who require effective interventions.

## 4 MATERIALS AND METHODS

### 4.1 Reagents and cell culture

We designed a series of pyrazolopyridine compounds in search of highly active and selective BTK inhibitors. After rigorous screening, we identified JDB175, which exhibited potent inhibitory activity against BTK (Figure 1A). Human lymphoma cell lines Namalwa, Raji, Ramos, and SU‐DHL‐4 were purchased from the American Type Culture Collection. The cells were cultured in RPMI 1640 medium supplemented with 10% fetal bovine serum and 1% penicillin/streptomycin (HyClone) in a 37°C incubator with 5% CO_2_. The Annexin V‐PE/7‐AAD double staining kit was purchased from BioLegand. Propidium iodide (PI), RIPA buffer, and proteinase inhibitor cocktail were obtained from Beyotime. D‐Luciferin potassium salt was purchased from Abcam.

### 4.2 Cell viability assay

The manufacturer's protocol was followed to conduct the cell viability assay. In brief, lymphoma cells were placed in 96‐well plates with a seeding density of 6 × 10^3^ to 2 × 10⁴ cells in 100 µL medium per well. Subsequently, 100 µL medium containing varying doses of JDB175 was added. After the designated treatment period, 20 µL of cell counting kit‐8 (CCK‐8) solution (MedChemExpress) was added to each well and incubated at 37°C for 2 h. The absorbance at 450 nm was measured using a microplate spectrophotometer (Molecular Devices) and the inhibition rate at each concentration was calculated. The IC_50_ values were calculated using GraphPad Prism 8 software (GraphPad Inc.).

### 4.3 Cell cycle and apoptosis analysis

After treatment with different doses of JDB175 for 48 h, the cells in six‐well plates were harvested in 75% ethanol for 24 hours. The cells were then stained with buffer containing 50 µg/mL PI, 50 µg/mL RNase A, and 0.1% Triton X‐100 for 30 min. Cell cycle distributions of the cells were analyzed by FCM with an ACEA NovoCyte flow cytometer and analyzed by NovoExpress software (ACEA Biosciences Inc.).

Following a 72‐h treatment with designated concentrations of JDB175, the cells were harvested and gently rinsed with phosphate‐buffered saline (PBS). Subsequently, they were individually stained with Annexin V‐PE and 7‐AAD for a 15 min incubation at room temperature, all under subdued light conditions. Cell apoptosis rates were assessed using an ACEA NovoCyte flow cytometer and subsequently analyzed with NovoExpress software.

### 4.4 Western blot analysis

After treatment with JDB175 for 48 h, the cells were harvested and protein lysates were prepared in RIPA buffer containing proteinase inhibitor cocktail. Subsequent western blot analyses were performed using the indicated primary antibodies and secondary antibodies conjugated to horseradish peroxidase. The protein bands were developed after incubating with chemiluminescent substrate (Amersham Biosciences). Quantification of the band intensities was based on three independent experiments using ImageJ software. Antibodies for western blot analysis were purchased from Cell Signaling Technology (anti‐AKT, 4685S, 1:1000; anti‐p‐γ‐H2AX, 9718S, 1:1000; anti‐cleaved caspase3, 9664S, 1:1000), ABclonal Technology (anti‐γ‐H2AX, A11463, 1:1000), and Abways (BTK, CY5733, 1:1000; p‐BTK, CY5558, 1:1000; p‐p44/42 MAPK, CY5044, 1:1000, p44/42 MAPK, AB3373, 1:1000; p‐AKT, CY6569, 1:1000; GAPDH, AB2000, 1:10,000; BAX, CY5059, 1:1000; cyclin D1, CY5404, 1:1000; CDK2, CY5020, 1:1000; p21, CY5543, 1:1000).

### 4.5 Distributions of JDB175 in the plasma and brain

After oral administration of JDB175, orelabrutinib, and SAR442168 at a dose of 10 mg/kg in female Sprague–Dawley rats, the rats were sacrificed and blood plasma and brain samples were collected 0.5 h later. Brain homogenate was prepared by homogenizing the tissue with 4 volumes (w:v) of a homogenizing solution consisting of methanol and 15 mM PBS in a 1:2 (v:v) ratio. Following protein precipitation, the concentration of the drugs in the brain and blood was analyzed using The Sciex QTRAP 6500 liquid chromatography–mass spectrometry/mass spectrometry System. The UPLC conditions were as follows: mobile phase A consisted of 0.1% formic acid (FA) in water, mobile phase B consisted of 0.1% FA in acetonitrile, the column used was ACQUITY UPLC HSS T3 1.8 µm 2.1 × 50 mm, the column temperature was maintained at 45°C, and the flow rate was set at 0.65 mL/min. The brain penetration rate of the drugs 0.5 h after oral administration was defined as the brain‐to‐plasma concentration ratio.

### 4.6 Establishment of CNS lymphoma model

The Institutional Animal Care and Use Committee of Sichuan University (Chengdu, China) approved all animal experiments (approval number: 2022120653). To establish a model of CNS lymphoma, female nude mice (8–10 weeks old) received intracarotid artery injections. A total of 5 × 10⁵ Namalwa cells expressing luciferase were injected into the internal carotid artery of each mouse using 100 µL of Hank's balanced salt solution. Three days after the surgery, the mice were randomly divided into groups and received daily oral administration of JDB175 (150 mg/kg) or vehicle. The solvent mixture for preparing JDB175 consisted of 5% dimethyl sulfoxide (DMSO), 10% Solutol, 40% polyethylene glycol 400 (PEG 400), and 45% ultrapure water. In vivo imaging was conducted 14 and 19 days after the model was established to observe the signal intensity of intracranial tumor cells.

### 4.7 Toxicity evaluation

After 30 days of oral administration of JDB175 (150 mg/kg), the mice were sacrificed and the heart, liver, spleen, lung, and kidney of the mice were collected and fixed in 4% paraformaldehyde followed by H&E staining for histopathological examination.

### 4.8 Statistical analyses

Data are represented as the mean ± standard deviation and processed by the GraphPad Prism 8 program. Comparisons between two groups were analyzed using Student's *t*‐test, and differences among multiple groups were analyzed using one‐way analysis of variance. A *p*‐value <0.05 was considered statistically significant.

## AUTHOR CONTRIBUTIONS

X.W. contributed to the design of the study. Y.X. and X.L. performed data acquisition, data analysis, and interpretation and drafted the manuscript. N.J. performed data acquisition and data analysis. X.W. performed manuscript revision. All authors approved the final version of the manuscript.

## CONFLICT OF INTEREST STATEMENT

Author Xiawei Wei and Ning Jiang are employees in Jumbo Drug Bank Co., Ltd. The other authors have no conflicts of interest to declare.

## ETHICS STATEMENT

All animal experiments were approved by the Institutional Animal Care and Use Committee of Sichuan University (approval number: 2022120653).

## Data Availability

The data included in this study are available upon request from the corresponding author.
